# Nurse-led self-management support after organ transplantation—protocol of a multicentre, stepped-wedge randomized controlled trial

**DOI:** 10.1186/s13063-021-05896-0

**Published:** 2022-01-06

**Authors:** Regina van Zanten, Monique van Dijk, Joost van Rosmalen, Denise Beck, Robert Zietse, Ann Van Hecke, AnneLoes van Staa, Emma K. Massey, On behalf of the aanZET study group, Denise Beck, Monique van Dijk, Marleen Goedendorp, Martijn van den Hoogen, Erwin Ista, Louise Maasdam, Olivier Manintveld, Emma K. Massey, Joost van Rosmalen, Annelies de Weerd, Regina van Zanten, Robert Zietse, Janet Been-Dahmen, AnneLoes van Staa, Ann Van Hecke, Jeannet Bisschop, Paul van der Boog, Maaike Konijn, Marjo van Helden, Luuk Hilbrands, Coby Annema, Lyda Engelsman, Tally Norder, Christina Oosterhoff, Irma Saro, Geesje Smeenge, Sanne Bosman, Arjan van Zuilen, Marleen van Buren, Marcia Kho, Marlies Reinders, Ruth Dam, Tessa van Diemen, Esther Nijgh, Esther de Haan, Anja Kooistra

**Affiliations:** 1 Department of Internal Medicine, Erasmus MC Transplant Institute, University Medical Center, Dr. Molewaterplein 40, Rotterdam, 3015 GD The Netherlands; 2 Department of Internal Medicine, Nursing Studies, Erasmus Medical Center, Rotterdam, The Netherlands; 3 Department of Biostatistics, Erasmus Medical Center, Rotterdam, The Netherlands; 4 Department of Epidemiology, Erasmus Medical Center, Rotterdam, The Netherlands; 5 Department of Public Health and Primary Care, University Centre for Nursing and Midwifery, Ghent University Hospital, Ghent, Belgium; 6 Department of Nursing Director, Ghent University Hospital, Ghent, Belgium; 7 University of Applied Science Rotterdam, Rotterdam, The Netherlands

**Keywords:** Nurse practitioners, Holistic nursing, Self-management, Patient participation, Behaviour mechanisms, Motivation, Goal, Self-efficacy, Self-control, Psychotherapy

## Abstract

**Background:**

Recipients of an organ transplantation face a number of challenges and often need to change their health behaviour. Good self-management skills are essential for optimal clinical outcomes. However, few interventions are available to support post-transplant self-management. To fill this gap, we developed a self-management support intervention offered by nurse practitioners. The primary aim of the study is to implement and test the effectiveness of the ZENN intervention in promoting self-management skills among heart, kidney liver and lung transplant recipients in comparison to standard care. The secondary aim is to assess the self-management support skills of nurse practitioners who will deliver the intervention.

**Methods:**

This multi-centre stepped-wedge randomized controlled trial will take place from September 2020 until May 2023. All departments will commence with inclusion of patients in the control period. Each department will be randomly assigned to a start date (step in the wedge) to commence the experimental period. Patients in the control period will receive standard care and will be asked to complete questionnaires at baseline (T0), 6 months (T1) and 12 months (T2), to assess self-management, self-regulation, quality of life and adherence. During the experimental period, patients will receive standard care plus the ZENN intervention and receive the same set of questionnaires as participants in the control period. Nurse practitioners will complete a baseline and follow-up questionnaire to assess differences in self-management support skills. Video recordings of outpatient clinic consultations during the control and experimental periods will determine the differences in nurses’ needs-thwarting and needs-supporting skills between the control and experimental period.

**Discussion:**

The ZENN intervention could be a useful approach to support patients’ self-management skills after organ transplantation and thus promote clinical outcomes as well as avoid adverse events.

**Trial registration:**

Dutch Trial Register NL8469. Registered on March 19, 2020.

**Supplementary Information:**

The online version contains supplementary material available at 10.1186/s13063-021-05896-0.

## Introduction

An organ transplantation is the preferred treatment for people with end-stage organ failure. However, after transplantation patients face several physical, social and emotional challenges. Patients need to deal with, for example, strict medication regimes, lifestyle changes and clinical appointments, social adjustment, and relationship instability [1–9]. They also have to cope with psychological consequences of transplantation such as acceptance of the organ, feelings towards the donor and changes in roles and relationships [10, 11]. Self-management can be defined as “the individual’s ability to manage the symptoms, treatment, physical and psychosocial consequences and lifestyle changes inherent with a chronic condition” [12]. In this definition, it is implied that self-management needs to be holistic in approach, whereby self-management of both medical and psychosocial aspects is required. Despite the scarcity of organs and the importance of effective self-management for patient outcomes, adherence to behavioural recommendations and therapeutic regimens after transplantation is substantial [4, 13, 14]. Optimizing patients’ self-management skills has received increasing attention, as this is essential for patient outcomes and can help reduce non-adherence [4, 13, 14], decrease healthcare costs [15] and improve patients’ quality of life [16].

Currently, there are few interventions to promote self-management among transplantation patients and these tend to focus mainly on medication adherence [17–19]. Moreover, these interventions have not been able to demonstrate effectiveness [18]. Patients after organ transplantation have specific needs in receiving self-management support, but the current healthcare system does not meet these needs [20–24]. Patients wish to discuss medical, social and emotional issues, receive information about their disease and receive tailored instructions [20].

Patient empowerment is also a crucial component of effective self-management. This concept can be defined as “a social process of recognizing, promoting and enhancing people’s abilities to meet their own needs, solve their own problems and mobilize the necessary resources in order to feel in control of their own lives” [25]. Patients indicate the need for support in becoming more self-confident and taking control over their post-transplant life, for example through positive feedback and care personalized to individual needs [21]. Linked to patient empowerment is the desire for shared-decision making. The extent to which patients want to participate in the decision-making varies [23], but there is a need for greater partnership between the patient and the professional. This partnership is a prerequisite for supporting self-management skills [21].

In clinical practice, physicians in outpatient clinics have limited time to address self-management issues and often lack the training in self-management support and empowerment skills to do so. Nurse practitioners (NPs) are more suitable to support patients in developing self-management skills and self-management support is an important part of the job description of nurses [26–28]. Still, nurses and NPs indicate the need for training to develop the necessary skills to support patients’ self-management [29, 30].

In response to the need for a more holistic, tailored and patient-centred approach to self-management support among patients and communication skills training among nurses, we initiated a project to develop a self-management support intervention. Based on previous studies of our team [20, 22, 23], we developed a self-management (support) intervention which is called the ZElfmanagement Na Niertransplantatie (ZENN; Dutch acronym for self-management after kidney transplantation) intervention [22]. This intervention included four key elements: (1) a general structure with room for individual tailoring, (2) a holistic approach, (3) shared-decision making between NP and patient, and (4) patient empowerment. The overall goal of the intervention is to enhance patients’ self-management skills in order to integrate their treatment- and life goals and subsequently optimize their quality of life and health-related outcomes.

In an initial qualitative pilot study among 26 kidney transplant recipients, we assessed the acceptability and feasibility of this intervention. Patients valued the ZENN intervention and felt that this should be offered to all patients who have received a kidney transplant, particularly in the first year post-transplant [31]. Moreover, professionals reported the added value of the holistic approach in building a relationship of trust and promoting well-being [31]. We concluded that it was worthwhile to test the effectiveness of the ZENN intervention in future research.

In this article (version 1; January 2021), we describe the design of the ‘aanZET’-study. In this study, we will test the effectiveness of the ZENN intervention in promoting self-management skills among heart, liver, lung and kidney transplant recipients in comparison to standard care. The aims of the study are (1) to assess if the intervention has an effect on patients’ self-regulation and self-management skills, quality of life, medication adherence, coping and self-efficacy, controlling for socio-demographic and medical characteristics; (2) to assess if these changes are sustained over time after completion of the intervention; and (3) to test if the training and delivering the intervention has an effect on self-management support skills among NPs who provide outpatient care after transplantation.

## Methods/design

### Study design and randomization

This multi-centre study in the Netherlands has an unblinded stepped wedge cluster randomized design [32] with pre- (T0) and post-intervention measures after six months (T1), with a follow up after twelve months (T2). The framework of this trial is superiority, whereby we will compare the outcomes between the control and intervention group, and between baseline and follow up data. Seven departments of five University Medical Centres will participate in the study, four kidney transplantation units, one heart transplantation unit, one liver transplantation unit and one lung transplantation unit. The coordinating centre is Erasmus MC, department Nephrology and Transplantation. This department is responsible for coordinating the study, distributing the questionnaires, training of the NPs and the data analysis. A steering committee is present to contribute ideas and to oversee the process.

**Fig. 1:**
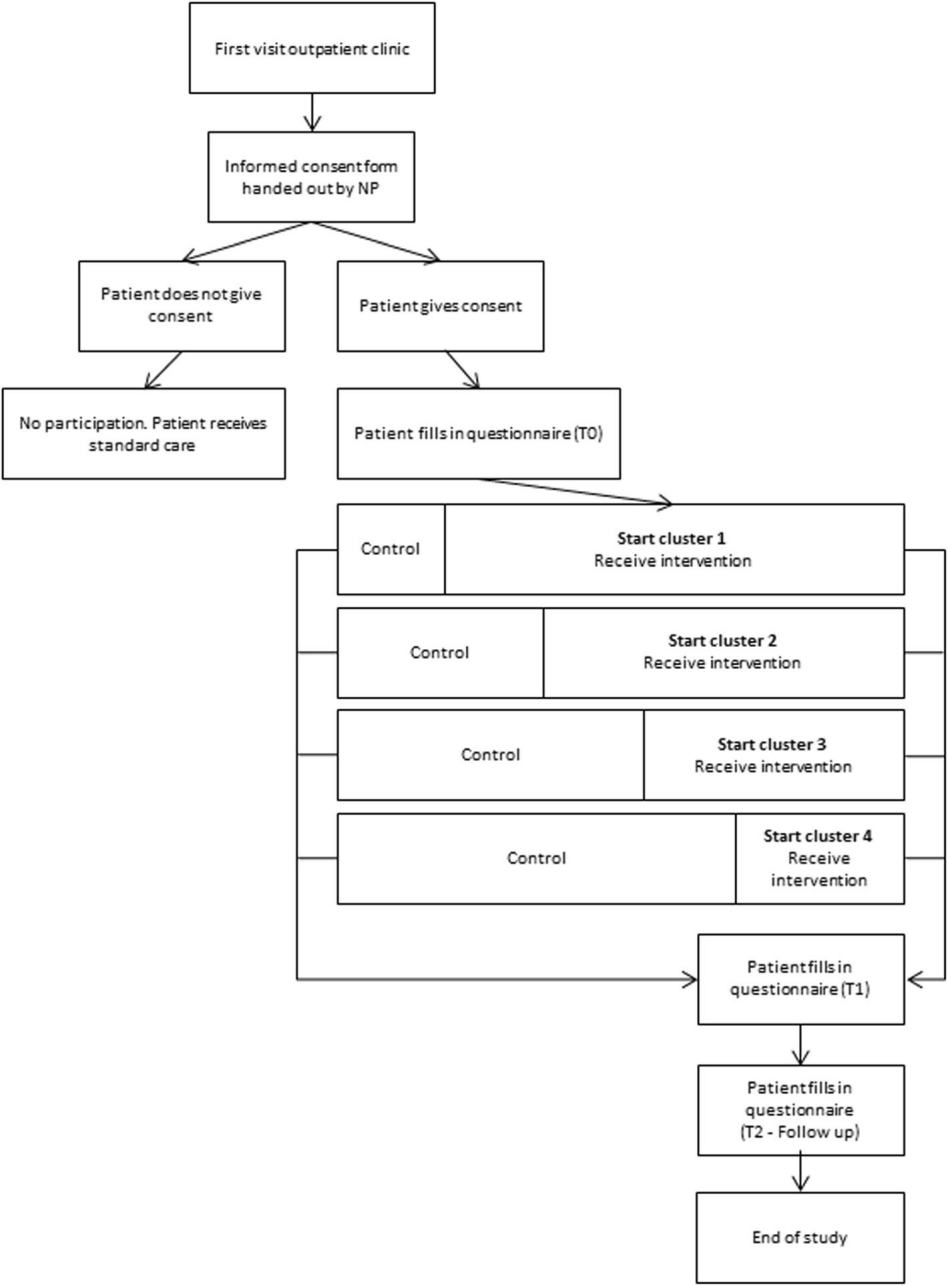
Flowchart of the study

Given the nature of the programme and the fact that NPs are trained in delivery of the intervention and communication skills, the NPs delivering the intervention cannot be blinded to the group allocation. Therefore, randomization will be performed at the department level. This will be carried out by an independent professional not involved in the study using sealed opaque envelopes. The envelopes with department names and envelopes with start date will be prepared and shuffled by the researcher. The independent professional then randomly chooses the envelopes. All seven participating departments commence the study with standard care i.e. the control period (see Fig. 1) in September 2020. The timing of transition of departments from control to experimental  period is randomized. The first department will be randomized to commence the experimental period in March 2021. During the experimental period, patients receive standard care plus the ZENN intervention. Patients in the control period are different from the experimental period patients, meaning that they will not cross-over.

In the month prior to the initiation of the experimental period for each department, the NPs will be trained in the delivery of the intervention. At this point in time, there will still be patients in the control period who are yet to complete the follow-up measure. During this month they may receive care in the form of a standard consultation from this NP. Therefore, there is potential for ‘contamination’ of these control period patients in the sense that their NPs will have already received the training associated with the intervention. To limit the risk of contamination, the following strategies will be put in place: standard care protocol will be used in all consultations for control period patients, and the questionnaires will not be administered by the NP but will be sent out by the researcher.

The ZENN intervention will be conducted in the outpatient clinic of each participating department. Due to the COVID-19 pandemic, the possibility of carrying out the intervention through tele-medicine is being examined. For the schematic overview of the inclusion, see Fig. 1. To enhance the validity of the study, the Standard Protocol Items: Recommendations for Interventional Trials (SPIRIT) guidelines [33] will be followed.

### Eligibility criteria

#### Patients

Eligible patients are heart, kidney, liver or lung transplant recipients who have received a transplant in one of the participating centres. The NP will ask patients to participate and hand out the written information on the study. Inclusion criteria are: over 18 years old, between two and thirteen months elapsed after transplantation, sufficient command of the Dutch language and the presence of a functioning graft. Exclusion criteria are cognitive limitations, participating in other lifestyle or self-management promoting programmes which could influence the outcome, and in case of kidney transplant recipients, an expected need for renal replacement therapy within 3 months of inclusion.

#### Nurse practitioners

NPs that give post-transplantation care at the outpatient clinic and have not been trained in the ZENN intervention before will be included.

### The intervention

The ZENN intervention [22] is based on the theoretical framework of the self-regulation theory [34]. The main intervention strategies are based on evidence-based techniques, namely goal setting and pursuit, Solution-Focused Brief-Therapy [35, 36] and Motivational Interviewing [37]. In practice, this means that the intervention focuses on a positive approach in order to enhance patients’ intrinsic motivation and self-efficacy to encourage sustainable behaviour change.

**Fig. 2:**
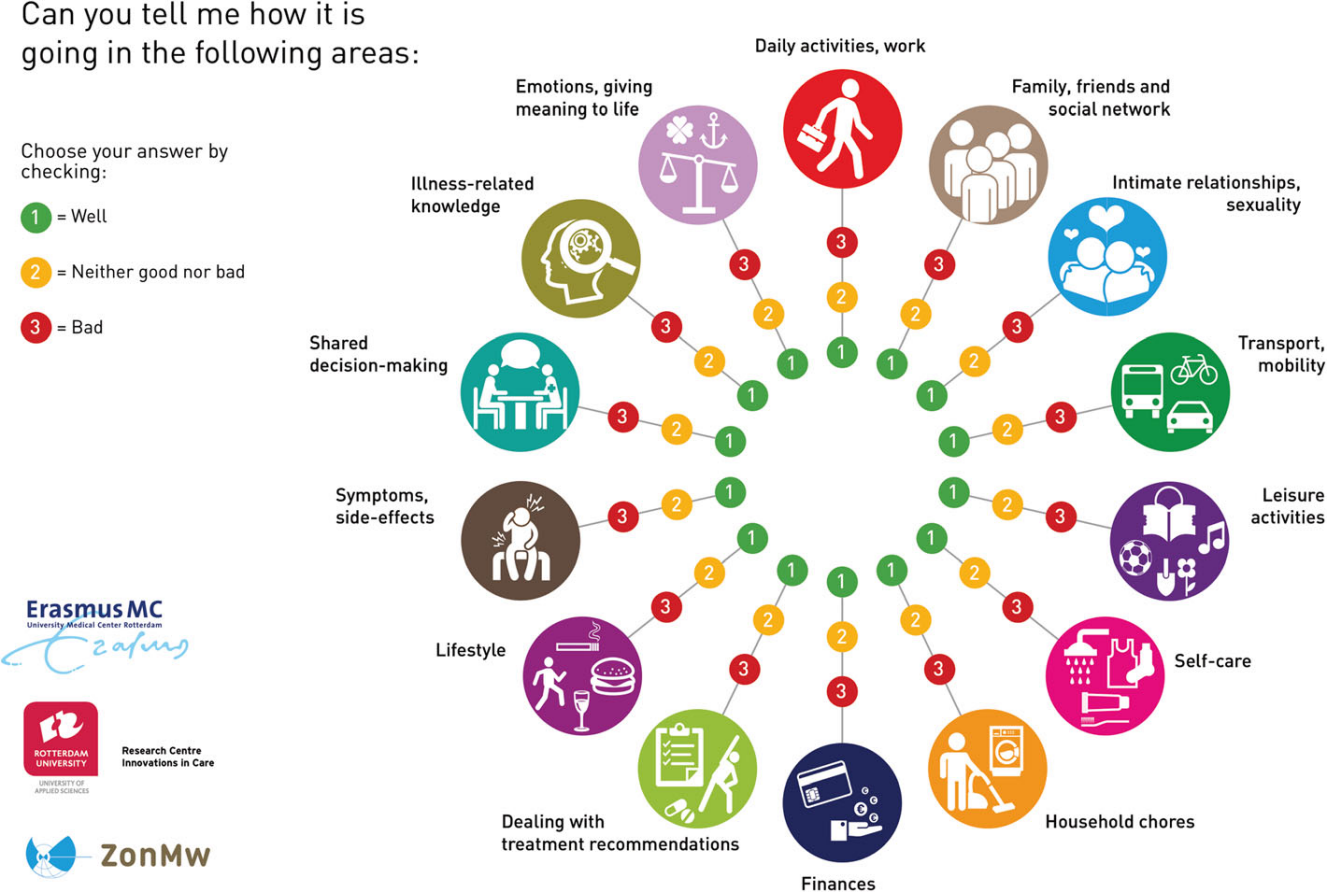
Self-management Web

The intervention is divided over several consultations, whereby the number of consultations depends on the logistical constraints of the setting and needs of the patient. In the initial session, a holistic review of how things are going in 14 life areas takes place, as outlined in the used communication aid, the Self-Management Web (see Fig. 2). The Self-Management Web is not a measurement tool, but is intended to structure the conversation, to open the view of both patient and NP to possible topics, and to make this visual for the patient. This web can also be revisited in subsequent sessions to make progress on personal goals visual and tangible. This tool is also suitable for patients who have lower health literacy or language skills [22].

**Fig. 3:**
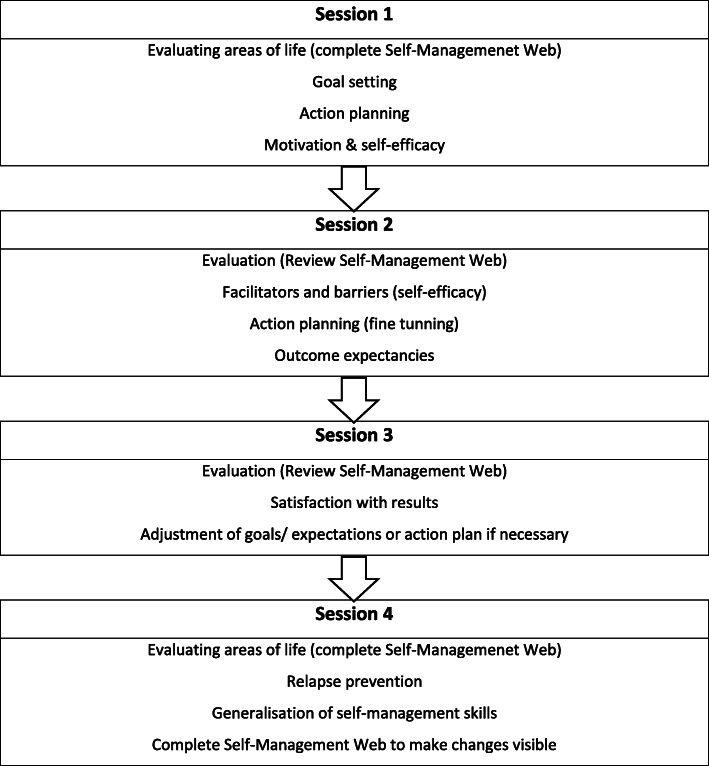
Content of sessions 1–4 [22]

After completing the Self-Management Web, the NP stimulates the patient to prioritize a life area and asks if he/she wants to address and set a SMART goal. A SMART goal is Specific, Measurable, Attainable, Relevant and Time-based. A global plan of action for goal attainment will be agreed upon. In addition, patients’ motivation for change and self-efficacy in relation to the goal will be discussed using visual analogue scales. The second and third session are used to evaluate the progress of goal attainment, facilitators and barriers are explored and if necessary, strengths emphasized and progress complimented, the action plan may be revised. In addition, motivation and self-efficacy will be evaluated and encouraged, alongside a discussion of internal versus external attribution of successes. This means that the patient will be stimulated to look at his/her own role in the success, to promote internal attribution of that success. The aim of the fourth session is to evaluate and discuss the goal attainment, relapse prevention and generalization of the learned skills to other situations. For a visual overview of the intervention see Fig. 3. The development of the ZENN intervention and its use has been extensively described elsewhere [22].

### Training

At the moment of transition to the experimental period, NPs will receive training in communication skills and intervention background by a psychologist (EM/DB). Both trainers were part of the team who developed the ZENN intervention. The content of the standardized training includes knowledge of the steps in the programme protocol, theoretical knowledge of behaviours change techniques, and practicing these techniques through role-play. NPs learn to activate patients to generate their own solutions rather than focusing on the problems and achieve progression towards personal goals in short space of time. The training consists of two blocks of three hours given on the same day. Due to the COVID-19 pandemic, the possibility of carrying out the training through e-learning and video conferencing is being examined. In addition, there is a booster session once implementation has begun with the aim of discussing cases and optimizing standardization between NPs. The experimental period starts immediately the day after the one-day training has been completed. An extensive description of the training according to the CRe-DEPTH criteria developed by Van Hecke et al. (2020) [38] can be found in Supplement A.

### Data collection and outcome measures

Patients who participate in the control or experimental period will be asked to complete online questionnaires at three points in time. Participants receive the questionnaires by email, or on paper, if requested. To promote completion of the follow-up during the control and experimental period, we will send participants two reminders if they have not completed the questionnaire.

#### Primary outcome measure—patients

The primary outcome measure is self-management skills, which will be assessed with the Skills and technique acquisition scale of the Health Education and Impact Questionnaire (heiQ) [39]. For an overview of the research questions and the used instruments, see Tables 1 and 2 in Appendix.

#### Secondary outcome measures—patients

##### Self-management skills

The 40-item Dutch Version of the heiQ [39] consists of eight domains, namely skills and technique acquisition which is the primary outcome (composite reliability 0.82), health directed activity (composite reliability 0.82), positive and active engagement in life (composite reliability 0.81), emotional distress (composite reliability 0.89), self-monitoring and insight (composite reliability 0.67), constructive attitudes and approaches (composite reliability 0.86), social integration and support (composite reliability 0.85), and health service navigation (composite reliability 0.85) [39]. Response options are based on a 4-point Likert scale: ‘Strongly disagree’ (1), ‘Disagree’ (2), ‘Agree’ (3), ‘Strongly agree’ (4). Interpretation of the heiQ is through mean scores on each domain, with subscale scores ranging between 1 and 4. Higher values on the domain indicate higher levels of self-management, with the exception of the scale ‘Emotional distress’, for which the interpretation is reversed.

##### Self-regulation in the context of transplantation

Currently available self-report measures of self-management among patients do not address all elements incorporated into the intervention and are generic. Therefore, a new self-report instrument based on the self-regulation theory [34] was developed tailored for the use in transplant recipients. An expert group and a group of kidney transplant patients assessed the item pool on relevance via the Content Validity Index [40] (expert group) and the Three-Step Test-Interview [41] (patients). The current item pool was scored as relevant, comprehensive and feasible, and therefore will be used in this study. The instrument consists of 47 items and assesses eight domains: awareness, attitude, self-efficacy, motivation, social support, skills, social comparison, and goal affect. With confirmatory factor analysis, we will establish the internal structure of this scale. Response options are ‘Strongly disagree’ (1), ‘Disagree’ (2), ‘Neither agree nor disagree’ (3), ‘Agree’ (4), ‘Strongly agree’ (5). Mean scores are calculated per domain. A higher score on the scale indicates a higher level of self-regulation skills in the context of a transplantation. We are in the process of testing the measurement properties which will be reported separately.

##### Quality of life

To assess quality of life (QoL), the 26-items World Health Organization Quality of Life – Brief Version (WHOQoL-BREF) [42] will be used. This instrument consists of five domains: physical health (Cronbach’s alpha = 0.80), psychological (Cronbach’s *α* = 0.74), social relationship (*α* = 0.66), environment (*α* = 0.73), and overall QoL and general health. Mean scores are calculated per domain as well as for the overall QoL. A higher score on the scale(s) indicates a higher level of QoL.

##### Medication adherence

Medication adherence will be assessed with the Basel Assessment of Adherence to Immunosuppressive Medication Scale (BAASIS©) [43]. The BAASIS is divided into two parts. The first part consists of four questions, with the answer options ‘Yes’ (1) and ‘No’ (0). If ‘Yes’, to any of these items, the second part needs to be answered; the patient will be asked to answer how often they are non-adherent: ‘Never’ (1), ‘Once a month’ (2), ‘Every 2 weeks’ (3), ‘Every week’ (4), ‘More than once a week’ (5), ‘Every day’ (6). When one of the items of the first part is answered with ‘Yes’, the patient is considered non-adherent.

#### Secondary outcome measures—nurse practitioners

##### Self-management support skills

The self-management support skills of the NPs before and after the experimental period will be evaluated using the Self-Efficacy and Performance in Self-Management Support (SEPSS-36) instrument [44]. The aim of the instrument is to assess self-efficacy and performance in self-management support. The SEPSS consists of 36 items, which are structured according to the Five-A’s model [45]. The items are divided into six subscales, namely assess (self-efficacy Cronbach’s *α* = 0.85; performance *α* = 0.84) , advise (self-efficacy *α* = 0.82; performance *α* = 0.75), agree (self-efficacy *α* = 0.89; performance *α* = 0.88), assist (self-efficacy *α* = 0.87; performance *α* = 0.85), arrange (self-efficacy *α* = 0.84; performance *α* = 0.82), and overall competencies (self-efficacy *α* = 0.83; performance *α* = 0.81). The response options for the items assessing self-efficacy are ‘Not at all’ (0), ‘Not sufficient’ (1), ‘More or less’ (2), ‘Sufficient’ (3), ‘Good’ (4). The items that assess performance can be scored with the following response options ‘Never’ (0), ‘Rarely’ (1), ‘Occasionally’ (2), ‘Frequently’ (3), ‘Always’ (4). Interpretation of the SEPSS is through the mean score on the subscale, ranging from 0 to 4. The scale total, self-efficacy and performance, will be calculated by summing the subscale scores, with a range from 0 to 24. A higher score indicates a higher level of self-efficacy or performance in self-management support.

##### Need-supportive counselling

The self-management support skills of the participating NPs will be objectively evaluated in the control and experimental period, via video observations using the Coding and Observing Need-Supportive Counselling in Chronic Care Encounters (COUNSEL-CCE) [46]. The patients and NPs who will be filmed, will be asked to give consent. Each NP will be filmed at least once in each period. The patients who are filmed will be a convenience sub-sample among participants. The aim of the instrument is to observe need-supportive and need-thwarting counselling in chronic care encounters. The COUNSEL-CCE (46) consists of 44 items divided into nine scales of behavioural approaches, namely participative (Cronbach’s *α* = .69), attuning (*α* = .88), guiding (*α* = .72), clarifying (*α* = .54), demanding (*α* = .78), domineering (*α* = .75), abandoning (*α* = .33), awaiting (*α* = .33), and relatedness supportive reciprocity (*α* = .81). Response options are based on a 4-point Likert scale range from ‘never observed’ (0) to ‘all the time observed’ (4). Procedure for the observation is: observation of the interaction between professional and patient during a 5-min interval, coding of the observed interval, observing the same interval for the second time, and coding any aspects missed. Before scoring the observations, the researchers will be trained in use of the manual, which is provided by the developers of the instrument. For each scale, a mean score will be calculated.

### Covariates

#### Patient-related

The following patient characteristics will be documented: *gender*, *age*, *educational level* (low, medium, high), *organ type* and *donor type (*deceased or living donation)*.* The question about donation will be completed by NP’s and not to by patients, due to the fact that patients are not always aware of the source of the organ.

#### Nurse practitioners-related

For NPs, we will record g*ender*, *age*, *educational level*, *years of experience as nurse practitioner*, *institute* and *department*.

### Intervention fidelity

The effect of the intervention will depend on adherence to the intervention protocol. The risk of not assessing the intervention fidelity is that it might be difficult to find out whether (non)significant findings are caused by how the intervention was administered or by the intervention itself [47]. Therefore, for each patient that receives the intervention, the NP will complete a questionnaire focusing on intervention fidelity. In this questionnaire, NP will be asked to answer questions about the number of consultations the patient received, how often the Self-Management web was used and if every step of the intervention was completed.

### Evaluation of experience

Participating patients will be asked about their experience with and the amount of nurse-led care on medical, emotional and social aspects, using questions included in the post-intervention measurement (T1). Overall experience of the nurse-led care will be assessed using a visual analogue scale and the Dutch translation of the subscale ‘patient-centeredness’ of the American Consumer Assessment of Health Plan Survey (CAHPS) [48]. We will use five questions of the subscale (Cronbach’s *α* = .83) [31].

Answer options for the three separate questions on the amount of the nurse-led care on medical, emotional and social aspects will be (0) ‘very little’ to (4) ‘very much’. Patients in the experimental period will also be asked about the experience with the intervention using open questions for experience with the intervention.

All participating NPs will be interviewed at the end of the experimental period about possible obstacles to implementation, room for improvement, advice for further development and implementation in clinical practice.

### Sample size

The sample size calculation is based on a power calculation for the primary outcome i.e. the Skills and Technique acquisition scale of the heiQ at T1. Previous studies have shown effect sizes (Cohen’s *d*) of similar interventions on the Skills and Technique Acquisition scale of 0.17, 0.50, 0.25, 0.50, 0.31 and 0.59 [49–54]. Based on these previous results, we estimate the effect size of the intervention to be 0.38, which is the average effect size observed in the previous studies. Using an ANCOVA model applied at the patient level, with an adjustment for baseline Skills and Technique Acquisition and treatment group (pre- or post-intervention), 82 patients per group are needed to obtain a power of 80% to detect a significant effect of the intervention, at a two-sided significance level of .05. To account for the effects of adjustment for covariates, dropout and missing data, and contamination, we aim to include 100 patients per group, 200 in total. Note that the ANCOVA model assumed in the power analysis differ slightly from the linear mixed model described for the primary analysis below.

### Data analysis

Data will be coded with a unique code per patient. The key of these codes will be safeguarded by the coordinating and the principal investigator with a password. No other persons will have access to the key. All data will be kept for 15 years, according to the Dutch Personal Data Protection law. Data will be stored using the data capture applications Gemstracker and Limesurvey. These systems are managed by the Department of Information Management of Erasmus MC and are secured with an individual password for the coordinating and principal investigator.

To assess if randomization has resulted in comparable groups, we will compare patients in the control and in the experimental period at baseline (T0) on characteristics as well as primary and secondary outcomes. Descriptive statistics will be presented as frequencies (%) for categorical variables. Continuous variables will be described as median and interquartile range for non-normally distributed variables and as mean and standard deviation for normally distributed data.

The primary analysis of the study is an initial univariate exploration of the effect of the intervention. Continuous outcomes at T1 between the patients in the control and experimental period will be compared and tested using the Mann-Whitney *U* test for non-normally distributed data and the independent-samples *t* test for normally distributed data. For the BAASIS, a 2 × 2 chi-squared test will be conducted (adherent yes/no × control/experimental group). In univariate analyses, the relationship between patient characteristics (such as age and gender) and medical characteristics (such as organ type and centre) and the outcomes will be assessed.

For the multivariate analyses, linear mixed models analysis will be conducted to account for repeated measures per patient, covariates organ and transplant centre and other significant covariates. All models will be estimated using intention-to-treat analysis incorporating all eligible patients from whom data was obtained. There are three measurement time points (T0, T1 and T2) to be included in these analyses. For missing data in predictor variables, we will use multiple imputation. A *p* value < 0.05 is considered statistically significant.

We will assess the differences on the primary and secondary outcomes between patients in the control period and versus the experimental period at both T1 and T2 on the outcomes (between-groups comparison). We will also assess the differences on the outcomes between T0–T1 and T0–T2 within the control period and within the experimental period (within-groups comparison). The secondary outcome measure *medication adherence*, which is a dichotomous variable, will be analysed with a generalized estimating equations logistic regression. If the effect of the intervention is found to differ according to organ type then separate explorative multivariate analyses will be conducted to assess the effect per organ.

Among NPs, in an initial univariate analysis, we will compare self-management support skills, using all scales of the SEPSS-36, before and after the experimental period (T0-prior to training and T1-at the end of the implementation period). For non-normally distributed data, we will use the Wilcoxon signed-rank test and the paired samples *t* test in case of normally distributed data.

The video recordings of the NPs will be assessed in blocks of 5 minutes each. Of every recording, a 5-minute clip will be randomly assigned to be observed by two researchers independently. The clips from the control period will be compared with the clips of the experimental period on the needs-thwarting and needs-supporting subscales and tested using the Wilcoxon-signed rank test for non-normally distributed data and the paired samples t-test in case of normally distributed data.

The interviews and qualitative questions in the questionnaire, which will be used to qualitatively evaluate the intervention and the nurse-led care, will be transcribed by at least two researchers independently and analysed thematically [55] using NVivo.

To examine if contamination occurred, we will compare the primary outcome (Skills and Technique Acquisition - heiQ) among control period patients in the contamination phase versus those in the control period not in the contamination phase at T1 and T2. This will be done using an independent samples *t* test. If there is a significant difference, we will control for contamination in the main multivariate analysis.

## Discussion

In the proposed multi-centre study, we will test the effect of the ZENN intervention (22) on organ transplant recipients, with a stepped-wedge clustered randomized controlled trial. It is also of interest to learn if the training to deliver the programme has an effect on NPs self-management support skills. This study was designed as a mixed-methods study, with a multidisciplinary research group.

### Strengths and limitations

Some strengths and weaknesses of the study design can be identified. The overall strength of the study is that we give patients in the experimental group the opportunity to participate in an intervention that may meet the needs of patients for holistic and tailored self-management support. Existing interventions have an insufficient tailoring to individual needs [18, 22, 56]; therefore, this intervention has the potential to make an important contribution to the lives of transplant recipients and approaches available to post-transplant professionals.

A strength of the stepped wedge design in this study is that the participating departments will be randomized so that, every department will have the opportunity to participate in the experimental period. With a classical randomization, whereby patients, NPs, or departments would be randomized into the control and the experimental arm, half of the patients would not receive the intervention or half of the NPs would not be trained. Randomization at the patient level is not possible due to the fact that nurses learn communication techniques and it is not possible to switch between these techniques during the control and experimental arm. Moreover, randomization at the NP level would be less preferable. Some departments have only one NP and if they will be randomized into the control arm, a whole department would not be able to participate in the intervention.

A limitation of the stepped wedge design is the risk of contamination of control period patients. Although the stepped wedge design eliminates to a great extent control-period patients being treated by a nurse trained in the intervention, there will be some patients at the end of the control period who are treated by and who undergo follow-up measurement once their NP has been trained. This means that it is possible that the patients at the end of the control period may be ‘contaminated’. We will register which control period patients fall into this ‘contamination phase’. We expect that this will be a small group and not be of influence on the findings.

A second potential limitation is that the effect on the patients is dependent on the effectiveness of the training in bringing change among the NPs. The fact that NP’s spend extra time with the patient could also increase patients’ self-management skills. To identify if an increase of patients’ self-management skills is affected by the intervention, we will conduct an evaluation of the intervention fidelity and ask patients to evaluate the programme.

Another potential limitation is response burden due to the amount of questionnaires patients’ need to complete. At each point in time, the patients are required to complete four questionnaires, which together comprise 117 questions and time to complete the questionnaires will be approximately 30 min. It has been suggested that shorter questionnaires can increase response rate and that respondents will be less likely to drop out and to complete the questionnaire if it takes 10 min compared to when it takes 30 min to complete [57]. Although there is a chance of response burden, we decided to accept this due to the fact that these questionnaires are the most appropriate to answer the research questions.

Another strength of the study is that we have broadened the target group to include other organ types, compared to the pilot study [22]. In the initial pilot study, the target group consisted of kidney transplant recipients, but we believe and research has shown, that self-management support is important for all transplant recipients regardless of the organ [13, 19, 20]. While the cause of organ failure differs, the post-transplant treatment regime, accompanying challenges and needs such as adjusting daily life, food hygiene and immunosuppression, but also psychosocial challenges are partly the same. Therefore, this study will include patients who received a heart, kidney liver or lung transplantation and still have a homogeneous study group. If found to be effective, we will have evidence to support implementation among all these transplant recipients.

## Trial status

Start of inclusion; September 2020, expected end of inclusion May 2022.

### Supplementary Information

**Additional file 1: Supplement A.**

**Additional file 2: Supplement B.**

## Data Availability

Not applicable, no datasets are included in this study protocol.

## Appendix

**Table 1:** Overview of research questions and measures at different time-points—patients

Research questions	Instrument	T0	T1	T2
**Patients in the control period**				
− What are the differences in patients’ self-management skills between control and experimental period	heiQ	X	X	X
− What are the differences in patients’ self-regulation skills in the context of transplantation?	Newly developed self-report instrument	X	X	X
− What is the difference in patients’ medication adherence between the control and experimental period?	BAASIS	X	X	X
− What is the differences in quality of life between patients in the control and experimental period?	WHOQoL-BREF heiQ	X	X	X
− What is the difference in patients’ experience and appreciation of nurse-led care?	Newly developed questions CAHPS		X	
**Patients in the experimental period**				
− What are the differences in patients’ self-management skills between the control and experimental period? − What are the differences in patients’ self-management skills between pre- and post-intervention?	heiQ	X	X	X
− What are the differences in patients’ self-regulation skills in the context of transplantation?	Newly developed self-report instrument	X	X	X
− What is the difference in patients’ medication adherence between patients in the control and experimental period? − What is the difference in patients’ medication adherence between the control and experimental period?	BAASIS	X	X	X
− What is the differences in quality of life after the transplantation between patients in the control and experimental period?	WHOQoL-BREF heiQ	X	X	X
− What is the difference in patients’ experience and appreciation of nurse-led care between the control and experimental period?	Newly developed questions CAHPS		X	
− What is patients’ experience and appreciation of the self-management intervention?	Newly developed questions		X	

**Table 2:** Overview of research questions and measures at different time-points—nurse practitioners

Research question	Instrument	T0	T1
− What are the differences in nurses’ self-management support skills between the control and experimental period?	SEPSS-36	X	X
− What are nurses’ experience and appreciation of the self-management intervention?	Semi-structured interview		X
− What is the difference in nurses’ needs-thwarting and needs supporting between the control and experimental period?	COUNSEL-CCE	X	X

## Abbreviations

BAASIS: Basel Assessment of Adherence to Immunosuppressive Medication Scale
COUNSEL-CCE: Coding and Observing Need-Supportive Counselling in Chronic Care Encounters
CAHPS: American Consumer Assessment of Health Plan Survey
heiQ: Health Education and Impact Questionnaire
NP: Nurse Practitioner
NURSE-CC: Nursing Research into Self-Management and Empowerment in Chronic Care
SEPPS-36: Self-Efficacy and Performance in Self-Management Support
WHOQoL-BREF: World Health Organization Quality of Life – Brief Version
ZENN: Zelfmanagement Na Niertransplantatie *Dutch acronym for* Self-Management after Kidney Transplantation
