# Supplementary material for: Nurse-led self-management support after organ transplantation—protocol of a multicentre, stepped-wedge randomized controlled trial
Source: Trials. 2022 Jan 6;23:14. doi: 10.1186/s13063-021-05896-0 (PMC8733435; doi:10.1186/s13063-021-05896-0)
Supplement: Supplementary file 1 — Additional file 1: Supplement A. [file 13063_2021_5896_MOESM1_ESM.pdf]

Supplement A

DEVELOPMENT OF THE TRAINING

1 Description of the aim or objectives of the training

Aims of the training are (1) to convey knowledge on the theoretical background of the intervention and the practical steps to be taken in intervention delivery and (2) to develop nurse practitioners (NPs) skills in communication techniques that are an essential part of the intervention. These aims were translated into learning objectives for NPs. After the training, NPs:

1. understand the positive, solution focused approach of the intervention
2. know the components/sessions of the intervention
3. know and understand the behavior change techniques incorporated in the intervention
4. be able to carry out the behavior change techniques incorporated in the intervention
5. feel confident in delivering the intervention

2 Description of the underlying theoretical framework of the training: the major theoretical framework underlying the training is that of Social Cognitive Theory [1]. This theory posits that behaviour can be learned or adapted through modelling, outcome expectancies, self-efficacy, and identification with the model. Social modelling (learning through observation of others) and mastery experiences (learning through own successful experience) will be deployed in order to further learning experiences during the training. These strategies will be operationalized as observation of video’s from the trainers performing a role-play, observation of other NPs in role-play, and participating in role-play.

3 Description of the developmental process: during intervention development using Intervention Mapping [2], multiple performance objectives were specified for NPs (as well as for patients). Performance objectives (PO) are behaviours the person must be able to carry out after the training (for successful intervention delivery). According to the Intervention Mapping methodology, we

subsequently assessed which determinants derived from behaviour change theories, such as Social Cognitive Theory, predict each PO. Based on the combination between POs and determinants, change objectives (CO) were developed. Change objectives describe the change in the determinant that predicts the desired behaviour. The manual was developed around these CO, incorporating strategies to achieve them. For an extensive explanation see Beck et al (2019)[3].

An example:

PO	NPs assess if recipients experience (self-management) challenges or problems in various areas of life
Determinant	Self-efficacy
Change objective	Feel confident about assessing and discussing challenges or problems in various areas of life

4 Description of target population and setting of the training: for this study the training was targeted at nurse practitioners (or nurse consultants) who offer care to transplant recipients in the out-patient clinic setting. This professional group was chosen as they are most often involved in supporting self-management in transplant centers.

5 Description of the educational resources: educational resources were developed by the project team. A presentation addressing the theoretic aspects as well as the practical steps of the intervention was developed. Video fragments demonstrating communication techniques were recorded. Also testimonials from NPs who had been trained and given the intervention previously were recorded and integrated in the programme. Furthermore, all participants receive an intervention manual specifying each step in the intervention and summarizing the theoretical underpinning of the intervention. Finally, an intervention checklist was developed to be completed by the nurse per patient. The purpose is to ensure each step has been carried out.

CHARACTERISTICS OF THE TRAINING

6 Description of the content of the training: during the training, knowledge is shared on the theoretical frameworks used in the intervention, namely Solution Focussed Brief therapy and Motivational Interviewing. The strategies incorporated into the intervention to attain each goal are explained. For example in order to gain a holistic view of the patients life and self-management challenges, the Self-management Web is administered. Another example is the use of ladders (from 0-10) to score current level of confidence, motivation, and progress towards goal attainment. These strategies are discussed and practiced.

7 Description of the format: the training is divided into two 3 hours blocks plus a booster session. Ideally there are 2-8 participants to create a comfortable environment for role-play. Block one consists of theoretical background and the structure of the intervention plus Session 1 of the intervention. Block two consists of sessions 2-4 of the intervention. The nurse is invited to participate in the subsequent training session (approximately 4-8 weeks later) as a booster once she has initiated implementation. The aim of this is to address any questions arising from the clinical experience and/or to practice her skills further.

8 Description of the didactic methods of training: as we aim to train all NPs so that they implement the intervention is the same way, the training is a protocol-centered training. Didactic elements are the transfer of knowledge. Interactive elements are the role-play and associated feedback. In line with Bigg's constructive alignment model of learning[4] we have specified learning objectives and developed teaching strategies to achieve these.

9 Description of tailoring of the training:

NPs receive written and audio-visual material to read prior to the first face-to-face session. They can submit questions to be addressed during the sessions. We have specified the learning objectives but

assess at the beginning of the training where each nurse feels they are in achieving this objective with a view to ensuring that each participant achieves each objective. For those participants who feel they have not reached the objectives, we will offer extra training.

CHARACTERISTICS OF THE TRAINERS

10 Description of the providers of the training: the trainers are two psychologists who developed the intervention[3]. Both psychologists are working in the field of transplantation and have hands-on experience with intervening with kidney transplant recipients. They have both delivered this training multiple times to groups of NPs[5].

ASSESSMENT OF TRAINING OUTCOMES

11 Description of the measured outcomes: as outlined in the study protocol, self-management support skills will be evaluated using the Self-Efficacy and Performance in Self-Management Support (SEPSS-36)[6] instrument before and after the intervention delivery period. After the intervention delivery period we will interview the NPs to assess the extent to which they felt they achieved the learning objectives. We will also ask their opinion of the training, changes in perceived competencies, behaviour and potential changes at the organisational level due to participation in the intervention-study. Additionally, to assess achievement of learning objective 4 we will make video observations of the NPs in the outpatient clinic before the training and afterwards (during implementation of the intervention) to assess self-management support skills. Observations will be coded according to the Coding and Observing Need-Supportive Counselling in Chronic Care Encounters (COUNSEL-CCE) instrument[7]. The training is a success when participants achieved the learning objectives.

Learning objective	Method	Instrument

**1.**	understand the positive, solution focused approach of the intervention	Interview	
**2.**	know the components/sessions of the intervention	Interview	
**3.**	know and understand the behavior change techniques incorporated in the intervention	Interview	
**4.**	be able to carry out the behavior change techniques incorporated in the intervention	Observation; Self-report	COUNCEL-CCE; SEPSS-36
**5.**	feel confident in delivering the intervention	Interview; Self-report	SEPSS-36

12 Description of the applied assessment method, including validity and reliability

Given the size of the group to be trained, we will use a mixed-methods approach. This will consist of (subjective) interviews described above combined with the (objective) COUNSEL-CCE to rate the observations. The COUNSEL-CCE is a tool to rate video clips in fragments of 5 minutes on 44 items divided over 2 dimensions of need-supportive or need-thwarting behaviours. Examples include 'the professional explores the patient's goals', and 'the professional puts pressure on the patient'. Items are scored on a 5-point Likert scale from never observed (0) to continuously observed (4). Internal construct validity and external validity have been demonstrated to be satisfactory[8]. Inter-rater reliability is generally good for need-supporting behaviours but lower for the need-thwarting behaviours.

References

1. Bandura A. Health Promotion by Social Cognitive Means. *Health Education & Behavior.* 2004;31(2):143-164.

2. Bartholomew LK PG, Kok G. *Planning health promotion programs : an intervention mapping approach.* San Francisco, California: Jossey-Bass; 2011.

3. Beck D, Been-Dahmen J, Peeters M, et al. A Nurse-Led Self-Management Support Intervention (ZENN) for Kidney Transplant Recipients Using Intervention Mapping: Protocol for a Mixed-Methods Feasibility Study. *JMIR Res Protoc.* 2019;8(3):e11856.

4. Biggs JB, & Tang, C. *Teaching for quality learning at university: What the student does.* New York: McGraw-Hill Education; 2011.

5. Been-Dahmen JMJ, Beck DK, Peeters MAC, et al. Evaluating the feasibility of a nurse-led self-management support intervention for kidney transplant recipients: a pilot study. *BMC nephrology.* 2019;20(1):143-158.

6. Duprez V, Van Hooft SM, Dwarswaard J, van Staa A, Van Hecke A, Strating MM. The development and psychometric validation of the self-efficacy and performance in self-management support (SEPSS) Instrument. *J Adv Nurs.* 2016;72(6):1381-1395.

7. Duprez V, Haerens L, Wuyts D, et al. Self-determination theory to observe healthcare professionals’ counselling during chronic care encounters: development of the COUNSEL-CCE tool. *Patient Educ Couns.* Online ahead of print:S0738-3991(0720)30666-30662.

8. Duprez V, Haerens L, Wuyts D, et al. Self-determination theory to observe healthcare professionals’ counselling during chronic care encounters: development of the COUNSEL-CCE tool. *Patient Educ Couns.* Submitted.
