# Supplementary material for: Nurse-led self-management support after organ transplantation—protocol of a multicentre, stepped-wedge randomized controlled trial
Source: Trials. 2022 Jan 6;23:14. doi: 10.1186/s13063-021-05896-0 (PMC8733435; doi:10.1186/s13063-021-05896-0)
Supplement: Supplementary file 2 — Additional file 2: Supplement B. [file 13063_2021_5896_MOESM2_ESM.pdf]

Supplement B - Schedule of enrolment, interventions, and assessments.

| | STUDY PERIOD | | | | |
|---|---|---|---|---|---|
| | **Enrolment** | **Allocation** | **Post-allocation** | | |
| **TIMEPOINT**** | | | ***$T_0$*** | ***$T_1$*** | *$T_2$* |
| **ENROLMENT:** | | | | | |
| **Eligibility screen** | X | | | | |
| **Informed consent** | X | | | | |
| **NP reports patient to researcher** | X | | | | |
| **Department allocation** | | X | | | |
| **INTERVENTIONS:** | | | | | |
| **Control group – care as usual** | | | | | |
| **Experimental group – Intervention** | | | ◆——— | ———◆ | |
| **ASSESSMENTS – <u>Patients in control group:</u>** | | | | | |
| **heiQ –** Self-management skills + QoL | | | X | X | X |
| **Newly developed self-report instrument –** <u>Self-regulation skills</u> | | | X | X | X |
| **BAASIS –** Medication adherence | | | X | X | X |
| **WHOQoL-BREF -** QoL | | | X | X | X |
| **newly developed questions -** Experience and appreciation of nurse-led care | | | | X | |
| **ASSESSMENTS – <u>patients in experimental group</u>** | | | | | |
| **heiQ –** Self-management skills + QoL | | | X | X | X |
| **Newly developed self-report instrument –** <u>Self-regulation skills</u> | | | X | X | X |
| **BAASIS –** Medication adherence | | | X | X | X |
| **WHOQoL-BREF -** QoL | | | X | X | X |
| **newly developed questions -** Experience and appreciation of nurse-led care | | | | X | |
| **Newly developed questions –** Experience and appreciation with intervention | | | | X | |
| **ASSESSMENT – <u>Nurse Practitioners</u>** | | | | | |
| **SEPSS-36 –** Self-management support skills | | | X | X | |

| | | | | | |
|---|---|---|---|---|---|
| **Semi-structured interview –** Experience and appreciation with intervention | | | | X | |
| **COUNSEL-CCE** – Nurses’ needs-thwarting and needs supporting | | | X | X | |
